# An unusual cause of painful ejaculation in a young patient: Zinner syndrome

**DOI:** 10.1016/j.amsu.2022.103982

**Published:** 2022-06-10

**Authors:** Moez Rahoui, Yassine Ouanes, Kays Chaker, Mokhtar Bibi, Kheireddine Mrad Dali, Ahmed Sellami, Sami Ben Rhouma, Yassine Nouira

**Affiliations:** Urology Department La Rabta Hospital, Tunis, Tunisia

**Keywords:** Zinner syndrome, Seminal vesical cyst, Renal agenesis

## Abstract

**Introduction:**

and importance: Zinner syndrome is a rare congenital malformation of the seminal vesicles and the homolateral upper urinary tract. While the majority of patients remain asymptomatic and are discovered incidentally, others present symptoms such as micturition or ejaculatory difficulties, or pain. We report a case of Zinner syndrome in a 32-year-old patient with painful ejaculation and discuss the diagnosis and treatment difficulties.

**Case presentation:**

A 32-year-old married patient was consulted for pelvic pain associated with painful ejaculation that had been evolving for six months. The clinical examination was normal. Routine laboratory studies of blood and urine were normal. The patient was explored by ultrasound which showed the absence of the right kidney and the presence of a 7 cm right lateral prostatic cystic mass. On MRI, the right kidney was not visualized. Multiple cysts were seen in the right seminal vesicle. Surgical excision of the cyst by laparotomy was performed. The patient had an uneventful recovery and was discharged on the third postoperative day.

**Clinical discussion:**

Congenital malformations of the seminal vesicles are often associated with those of the ipsilateral upper urinary tract, as the ureteral and seminal vesicle buds originate from the mesonephric duct. The syndrome often occurs in the second and third decades of life, especially after the onset of sexual activity. The most common symptoms were dysuria, perineal pain, epididymitis, and painful ejaculation. Diagnostic modalities include ultrasound, MRI, and cystoscopy. In patients with symptoms, the therapeutic management of the cyst includes ultrasound-guided aspiration and laparoscopic or open surgical excision.

**Conclusion:**

Seminal vesicle cysts associated with homolateral renal agenesis or hypoplasia are a rare urologic anomaly. The treatment depends on the patient's symptoms. surgical excision of seminal vesicle cysts may be needed for large cysts causing obstructive symptoms.

## Highlights

• Zinner syndrome is a rare congenital malformation of the seminal vesicles and the homolateral upper urinary tract.
• The most common symptoms were dysuria, perineal pain, epididymitis, and painful ejaculation.
• Diagnostic modalities include ultrasound, MRI, and cystoscopy.
• Surgical excision of seminal vesicle cysts may be needed for large cysts causing obstructive symptoms.

## 1 Introduction

First described in 1914, Zinner syndrome is a rare congenital malformation of the seminal vesicles and the homolateral upper urinary tract [1]. It is a rare condition with less than 200 cases reported in the literature [1]. It is usually detected in the third to fourth decade of life [2]. While the majority of patients remain asymptomatic and are discovered incidentally, others present symptoms related to seminal vesicle cysts or ejaculatory duct obstruction: micturition or ejaculatory difficulties or pain [2]. We report a case of Zinner syndrome in a 32-year-old patient with painful ejaculation and discuss the diagnosis and treatment difficulties. The work has been reported in line with the SCARE 2020 criteria [3].

## 2 Case report

**Fig. 1:**
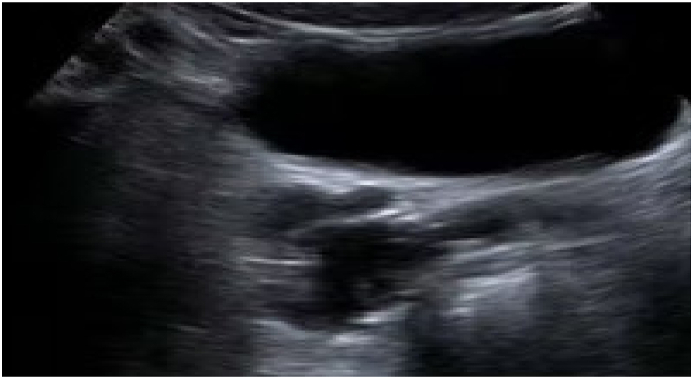
Ultrasound showing a cystic mass of the right seminal vesicle.

**Fig. 2:**
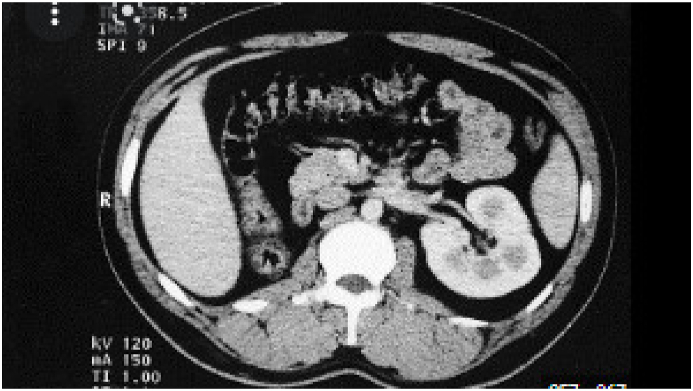
CT scan confirming right renal agenesis.

**Fig. 3:**
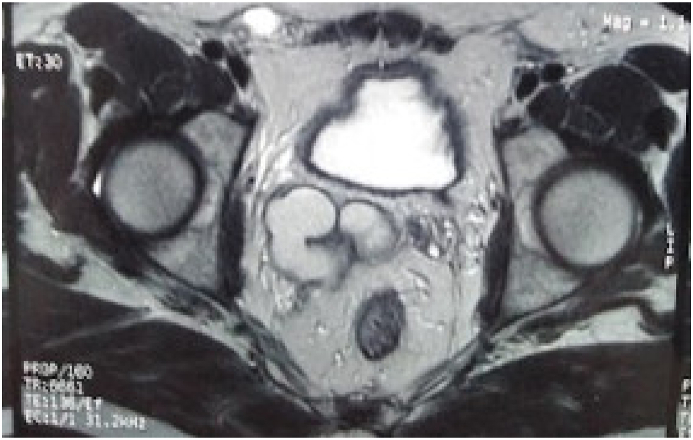
MRI showing the cyst of the right seminal vesicle.

A 32-year-old married patient was consulted for pelvic pain associated with painful ejaculation that had been evolving for six months. The clinical examination was normal. The digital rectal examination showed the presence of a soft mass at the right base of the prostate. Routine laboratory studies of blood and urine were normal. The patient was explored by ultrasound which showed the absence of the right kidney and the presence of a 7 cm right lateral prostatic cystic mass (Fig. 1). The CT scan confirmed the right renal agenesis and showed that the cystic mass was dependent on the right seminal vesicle (Fig. 2). To better explore the cystic nature of the mass, an abdominopelvic MRI was performed. On MRI, the right kidney was not visualized. Multiple cysts were seen in the right seminal vesicle, which followed signal intensity of simple fluid, being hypointense on T1-weighted sequences and hyperintense on T2-weighted sequences (Fig. 3). Based on the above findings of right renal agenesis and ipsilateral seminal vesicle cysts, a diagnosis of Zinner syndrome was made. After multidisciplinary consultation and discussion with the patient, surgical treatment was indicated. Surgical excision of the cyst by laparotomy was performed. The patient had an uneventful recovery and was discharged on the third postoperative day. After six months of clinical and radiological check-ups, there was no functional complaint.

## 3 Discussion

Congenital malformations of the seminal vesicles are often associated with those of the ipsilateral upper urinary tract, as the ureteral and seminal vesicle buds originate from the mesonephric duct [1,2]. This duct is a related organ in humans during embryogenesis. In men, it will give rise to hemi trigon, bladder neck, urethra, seminal vesicle, vas deferens, epididymis, and epididymal heads under the influence of testosterone and *anti*-Mullerian hormone [2]. Thus, any aggression in the first trimester could synchronously alter the embryogenesis of the kidney, ureter, seminal vesicle, and vas deferens [2]. The syndrome often occurs in the second and third decades of life, especially after the onset of sexual activity. The most common symptoms were dysuria, perineal pain, epididymitis, and painful ejaculation [4]. Our patient presented with pelvic pain and painful ejaculation. Diagnostic modalities include ultrasound, MRI, and cystoscopy. Ultrasound, especially transrectal ultrasound, is the most commonly used tool for the evaluation of seminal cysts and will reveal a pelvic cystic lesion unless superinfection is present [4]. MRI not only provides a definitive diagnosis of the cystic component resulting in a lesion with low signal intensity on the T1-weighted image and high signal intensity on the T2-weighted image; it is also of great help for surgical decision and approach [2,5]. Cystoscopy could show an incomplete trigone with extrinsic compression due to the mass effect of a large seminal vesicle cyst [5]. Simple monitoring is recommended in asymptomatic patients. In patients with symptoms, the therapeutic management of the cyst includes ultrasound-guided aspiration and transurethral detachment of the ejaculatory duct and seminal vesicular cyst [2,4]. Currently, laparoscopic surgery seems to be the most appropriate for surgical treatment of seminal vesicle cysts [6]. It has the advantage of direct access to the seminal vesicle with excellent depth imaging compared to open surgery. Removal of the seminal vesicle cysts can resolve symptoms while preserving fertility and erectile function [2,6]. Our patient has an open surgical excision of a cyst. After six months of clinical and radiological check-ups, there was no functional complaint.

## 4 Conclusion

Seminal vesicle cysts associated with homolateral renal agenesis or hypoplasia are a rare urologic anomaly. The complementary role of various imaging modalities is useful to confirm the diagnosis. The treatment depends on the patient's symptoms. While asymptomatic patients may be followed up with conservative management, surgical excision of seminal vesicle cysts may be needed for large cysts causing obstructive symptoms.

## Ethical approval

Not applicable.

## Sources of funding

This research did not receive any specific grant from funding agencies in the public, commercial, or not-for-profit sectors.

## Author contributions

Rahoui Moez, Ouannes Yassine and Chaker Kays: Data collection, Manuscript writing, Results discussion.

Bibi Mokhtar, Mourad Daly Kheireddine and Sellami Ahmed: Manuscript writing and revision.

Ben Rhouma Sami and Nouira Yassine: Paper revision.

## Registration of research studies

Name of the registry: N/a.

Unique identifying number or registration ID: N/a.

Hyperlink to your specific registration (must be publicly accessible and will be checked): N/a.

## Guarantor

Rahoui Moez is the guarantor of the study and accept full responsibility for the work and/or the conduct of the study, had access to the data and controlled the decision to publish.

## Consent

Written informed consent was obtained from the patient for publication of this case report and accompanying images. A copy of the written consent is available for review by the Editor-in-Chief of this journal on request.

## Provenance and peer review

Not commissioned, externally peer-reviewed.

## Declaration of competing interest

Authors do not report any conflict of interest.
